# Wireless technologies for the connectivity of the future

**DOI:** 10.1186/s13638-021-01980-w

**Published:** 2021-05-06

**Authors:** Filipe D. Cardoso, Vlatko Lipovac, Luis M. Correia

**Affiliations:** 1 ESTSetúbal, Polytechnic Institute of Setúbal and INESC-ID, Setúbal, Portugal; 2 University of Dubrovnik, Dubrovnik, Croatia; 3 IST/INESC-ID, University of Lisbon, Lisbon, Portugal

## Abstract

This Special Issue originates from the international conference EuCNC 2020 (European Conference on Networks and Communications), which was planned to be held in June 2020 in Dubrovnik (Croatia), but due to the COVID-19 pandemic was changed to an Online Conference. The Technical Programme Chairs of the conference have selected the best papers and invited authors to submit an extended version of their paper, by at least one third of their length. Only the top ranked papers were invited to this Special Issue, in order to fulfil its purpose. The main target was to collect and present quality research contributions in the most recent activities related to systems and networks beyond 5G, already presenting ideas for 6G. Through this Special Issue, the state-of-the-art is presented and the new challenges are highlighted, regarding the latest advances on systems and network perspectives that are already being positioned beyond 5G, bridging as well with the evolution of 5G, including applications and trials. Therefore, the motivation for this Special Issue is to present the latest and finest results on the evolution of research of mobile and wireless communications, coming, but not exclusively (since EuCNC is a conference open to the whole research community), from projects co-financed by the European Commission within its R&D programmes.

5G system aspects have already entered the commercial phase, and its networks ones (in the so-called Stand-Alone approach) will soon reach this phase. This means that, from the research viewpoint, work has started to address what will not be implemented in 5G, not only extending concepts for further phases of 5G but also initiating the views on what 6G may be composed of. From the viewpoint of backward compatibility, it is important that 6G potential technologies may bridge with 5G ones, but it is also necessary that new concepts are put forward. In fact, there are already appearing coordinated activities and projects in some countries addressing 6G, which shows the importance of this topic. Some current topics remain important, e.g., new frequency bands (extending beyond mm waves), mobile IoT, softwarisation of networks, network architectures, wearables and bridging with body area networks, security and privacy, and artificial intelligence in communications, among others, but one can expect that new ones are proposed. The scope of this Special Issue is to focus on new approaches beyond 5G, but still bridging with the foreseeable implementation of 5G.

In what follows, one presents a brief overview of the papers composing the current Special Issue.

*End-to-End Network Slice Architecture and Distribution Across 5G Micro-Operator Leveraging Multi-Domain and Multi-Tenancy*, authored by Idris Badmus, Abdelquoddouss Laghrissi, Marja Matinmikko-Blue and Ari Pouttu, addresses a sophisticated end-to-end network slicing architecture for different deployment scenarios of the local 5G micro-operator concept. Local 5G networks are emerging as a new form for 5G deployment, targeting service delivery for vertical-specific purposes and other local users. These networks are also known as micro-operator networks for which prior work has established different deployment scenarios, namely Closed, Open and Mixed Networks. To achieve network flexibility, customization and privacy required by various vertical sectors, such as industry, health and energy, it is essential to have a well-defined network slicing architecture and adequate implementation procedure. In this paper, a sophisticated end-to-end network slicing architecture is proposed for different deployment scenarios of the local 5G micro-operator concept. The proposed architecture incorporates a broad four-layer concept, leveraging a multi-tenancy layer for different tenants and their end users, a descriptive service layer, a multi-domain slicing Management and Orchestration (MANO) layer, and a resource layer. The authors further propose a network slice instance (NSI) communication service distribution technique for local 5G micro-operators. This is achieved by expanding/leveraging the communication service management function (CSMF) in the multi-tenant layer into a multi-tenant manager and an orchestrator of communication services. In addition, it is described how the communication service orchestrator will address all the possible multitenant-slice situations during the distribution of a network slice instance to multiple tenants. The novel methods described in the paper present a solution for not only network slice communication service distribution across different micro-operator’s tenants but also for future use cases, especially, when the allocated slice is responsible for multiple tenants or when a tenant request multiple NSIs.

*Actor-Critic Learning based Energy Optimization for UAV Access-and-Backhaul Networks*, authored by Yaxiong Yuan, Lei Lei, Thang X. Vu, Symeon Chatzinotas, Sumei Sun and Björn Ottersten, addresses energy minimization with a limited power supply for both backhaul and access links in unmanned aerial vehicle (UAV)-assisted networks. The UAV acts as an aerial base station which acquires the requested data via backhaul link and then serves ground users (GUs) through an access network. In this paper, the authors investigate an energy minimization problem with a limited power supply for both backhaul and access links. The difficulties for solving such a non-convex and combinatorial problem lie at the high computational complexity/time. In solution development, the approaches from both actor-critic deep reinforcement learning (AC-DRL) and optimization perspectives are considered. Firstly, two offline non-learning algorithms, i.e., an optimal and a heuristic algorithms, based on piecewise linear approximation and relaxation are developed as benchmarks. Secondly, towards real-time decision making, the conventional AC-DRL are improved and two learning schemes: AC-based user group scheduling and backhaul power allocation (ACGP), and joint AC-based user group scheduling and optimization-based backhaul power allocation (ACGOP) are proposed. Numerical results show that the computation time of both ACGP and ACGOP are reduced tenfold to 100-fold compared to the offline approaches, and ACGOP is better than ACGP in energy savings. The results also verify the superiority of proposed learning solutions in terms of guaranteeing the feasibility and minimizing the system energy compared to the conventional AC-DRL.

*An Experimental Publish-Subscribe Monitoring Assessment to Beyond 5G Networks*, authored by Ramon Perez, Jaime Garcia-Reinoso, Aitor Zabala, Pablo Serrano and Albert Banchs, is focused on fifth-generation (5G) of mobile networks designed to accommodate different types of use cases, each of them with different and stringent requirements and key performance indicators (KPIs). To support the optimization of the network performance and validation of the KPIs, there exists the necessity of a flexible and efficient monitoring system, capable of realizing multi-site and multi-stakeholder scenarios. Nevertheless, for the evolution from 5 to 6G, the network is envisioned as a user-driven, distributed Cloud computing system where the resource pool is foreseen to integrate the participating users. In this paper, the authors present a distributed monitoring architecture for Beyond 5G multi-site platforms, where different stakeholders share the resource pool in a distributed environment. Taking advantage of the usage of publish-subscribe mechanisms adapted to the Edge, the developed lightweight monitoring solution can manage large amounts of real-time traffic generated by the applications located in the resource pool. The performance of the implemented paradigm was accessed, revealing some interesting insights about the platform, such as the effect caused by the throughput of monitoring data in performance parameters such as the latency and packet loss, or the presence of a saturation effect due to software limitations that impacts in the performance of the system under specific conditions. In the end, the performance evaluation process has confirmed that the Monitoring platform suits the requirements of the proposed scenarios, being capable of handling similar workloads in real 5G and Beyond 5G scenarios, then discussing how the architecture could be mapped to these real scenarios.

*Synchronization in 5G Networks: a Hybrid Bayesian Approach towards Clock Offset/Skew Estimation and Its Impact on Localization*, authored by Meysam Goodarzi, Darko Cvetkovski1, Nebojsa Maletic, Jesús Gutiérrez and Eckhard Grass, addresses clock synchronization in 5G networks, a major challenge when designing wireless networks. This paper focuses on tackling the time synchronization problem in 5G networks by adopting a hybrid Bayesian approach for clock offset and skew estimation. Furthermore, we provide an in-depth analysis of the impact of the proposed approach on a synchronization-sensitive service, i.e., localization. Specifically, the authors expose the substantial benefit of Belief Propagation (BP) running on Factor Graphs (FGs) in achieving precise network-wide synchronization. Moreover, the proposed approach takes advantage of Bayesian Recursive Filtering (BRF) to mitigate the time-stamping error in pairwise synchronization. Finally, the merit of hybrid synchronization by dividing a large-scale network into local synchronization domains and applying the most suitable synchronization algorithm (BP- or BRF-based) on each domain is revealed. The performance of the hybrid approach is then evaluated in terms of the Root Mean Square Errors (RMSEs) of the clock offset, clock skew, and the position estimation. According to the simulations, despite the simplifications in the hybrid approach, RMSEs of clock offset, clock skew, and position estimation remain below 10 ns, 1 ppm, and 1.5 m, respectively.

*Outage Prediction for Ultra-Reliable Low-Latency Communications in Fast Fading Channels*, authored by Andreas Traßl, Eva Schmitt Tom Hößler, Lucas Scheuvens, Norman Franchi, Nick Schwarzenberg and Gerhard Fettweis, deals with outage prediction approaches for Rayleigh and Rician fading channels. The addition of redundancy is a promising solution to achieve a certain Quality of Service (QoS) for ultra-reliable low-latency communications (URLLC) in challenging fast fading scenarios. However, adding more and more redundancy to the transmission results in severely increased radio resource consumption. Monitoring and prediction of fast fading channels can serve as the foundation of advanced scheduling. By choosing suitable resources for transmission, the resource consumption is reduced while maintaining the QoS. In this article, the authors present outage prediction approaches for Rayleigh and Rician fading channels. Appropriate performance metrics are introduced to show the suitability for URLLC radio resource scheduling. Outage prediction in the Rayleigh fading case can be achieved by adding a threshold comparison to state-of-the-art fading prediction approaches. A line of sight (LOS) component estimator is introduced that enables outage prediction in LOS scenarios. Extensive simulations have shown that under realistic conditions, effective outage probabilities of 10–5 can be achieved while reaching up-state prediction probabilities of more than 90%. We show that the predictor can be tuned to satisfy the desired trade-off between prediction reliability and utilizability of the link. This enables our predictor to be used in future scheduling strategies, which achieve the challenging QoS of URLLC with fewer required redundancy.

*A Study About Signal Variation with Minor Receiver Displacement in a Meeting Room at 60 GHz: Measurements and Simulations*, authored by Muhammad Usman Sheikh, Kalle Ruttik, Riku Jäntti and Jyri Hämäläinen, aims to study the impact of small receiver displacement on a signal propagation in a typical conference room environment at a millimeter wave (mmWave) frequency of 60 GHz. While channel measurements provide insights on the propagation phenomena, their use for the wireless system performance evaluation is challenging. Whereas, carefully executed three-dimensional ray tracing (RT) simulations represent a more flexible option. Nevertheless, a careful validation of simulation methodology is needed. The first target of this article is to highlight the benefits of an in-house built three-dimensional RT tool at 60 GHz, and shows the effectiveness of simulations in predicting different characteristics of the channel. To validate the simulation results against the measurements, two different transmitter (Tx) positions and antenna types along with ten receiver (Rx) positions are considered in a typical conference room. In first system configuration, an omnidirectional antenna is placed in the middle of the table, while in the second system configuration a directed horn antenna is located in the corner of the meeting room. After validating the simulation results with the measurement data, in the second part of this work, the impact of a small change i.e., 20 cm in the receiver position is studied. To characterize the impact the authors apply as performance indicators the received power level, root mean square delay spread (RMS-DS), and RMS angular spread (RMS-AS) in azimuth plane. The channel characteristics are considered with respect to the direct orientation (DO) i.e., the Rx antenna is directed towards the strongest incoming path. Different antenna configurations at the Tx and Rx side are applied to highlight the role of antenna properties on the considered channel characteristics. Especially, in the second system configuration the impact of different antenna half power beamwidth on different considered channel characteristics.

is highlighted through acquired simulation results. The validation of results shows the RMS error of only 2–3 dB between the measured and simulated received power levels for different Tx configurations in the direction of DO. Results indicate that only a small change of the Rx position may result a large difference in the received power level even in the presence of line-of-sight between the Tx and Rx. It is found that the STD of received power level across the room increases with the decrease in HPBW of the antenna. As can be expected, directed antennas offer lower value of RMS-DS and RMS-AS compared with isotropic antenna.

*CARAMEL: Results on a Secure Architecture for Connected and Autonomous Vehicles Detecting GPS Spoofing Attacks*, authored by Christian Vitale, Nikos Piperigkos, Christos Laoudias, Georgios Ellinas, Jordi Casademont et al., addresses the cybersecurity gaps introduced by the new technological domains adopted by modern vehicles applying, among others, advanced Artificial Intelligence and Machine Learning techniques. As a result, the H2020-CARAMEL enhances the protection against threats related to automated driving, smart charging of Electric Vehicles, and communication among vehicles or between vehicles and the roadside infrastructure. This paper focuses on the latter and presents the CARAMEL architecture aiming at assessing the integrity of the information transmitted by vehicles, as well as at improving the security and privacy of communication for connected and autonomous driving. The proposed architecture includes: (1) multi-radio access technology capabilities, with simultaneous 802.11p and LTE-Uu support, enabled by the connectivity infrastructure; (2) a MEC platform, where, among others, algorithms for detecting attacks are implemented; (3) an intelligent On-Board Unit with anti-hacking features inside the vehicle; (4) a Public Key Infrastructure that validates in real-time the integrity of vehicle’s data transmissions. As an indicative application, the interaction between the entities of the CARAMEL architecture is showcased in case of a GPS spoofing attack scenario. Adopted attack detection techniques exploit robust in-vehicle and cooperative approaches that do not rely on encrypted GPS signals, but only on measurements available in the CARAMEL architecture.

*Cooperative Non-Orthogonal Multiple Access for Wireless Communication Networks by Exploiting the EXIT Chart Analysis*, authored by Zeyad Elsaraf, Abbas Ahmed, Faheem Ahmad Khan, and Qasim Zeeshan Ahmed, addresses Successive Interference Cancellation (SIC) in Non-Orthogonal Multiple Access (NOMA). In the next generation of mobile communication networks, unprecedented challenges are required to be met, such as, much higher data rates and spectrum efficiency, lower latency, and massive connectivity. NOMA has recently been proposed as a promising technology to achieve much superior spectral efficiency compared to conventional orthogonal multiple access techniques employed in present communication systems. A salient feature of NOMA is its use of SIC to decode users’ information when multiple users are allowed to transmit in same time/frequency/code domain. In this paper, the authors aim to exploit an aspect of SIC, namely the availability of other users’ data to realize a cooperative NOMA system. EXtrinsic Information Transfer (EXIT) charts are utilized to examine the performance of proposed system in terms of user fairness while employing IRregular Convolutional Codes (IRCC)s. The EXIT chart using IRCC evaluates the convergence analysis for the proposed system. Further, to evaluate the system performances in cooperative NOMA system, the authors have derived the expressions for the achievable rates which are obtained independently and utilized them in evaluating the experimental data for the proposed NOMA model.

